# Seed‐Source Precipitation Drives Drought Acclimatization of *Reaumuria songarica*: Implications for Seed Germination and Seedling Growth in the HeHuang Valley

**DOI:** 10.1002/ece3.72010

**Published:** 2025-08-24

**Authors:** XinYou Wang, Lijun Zhang, Zhengshegn Li, Yanlong Wang, Ying Liu, YuShou Ma

**Affiliations:** ^1^ Qinghai Academy of Animal and Veterinary Sciences, Qinghai Provincial Key Laboratory of Adaptive Management on Alpine Grassland, Key Laboratory of Superior Forage Germplasm in the Qinghai‐Tibetan Plateau Qinghai University Xining Qinghai China; ^2^ College of Bioligical Sciences and Technology Yili Normal University Xinjiang China

**Keywords:** HeHuang Valley, optimal seed harvesting period, *Reaumuria songarica*, seed germination characteristics, seed trait, seedling growth state

## Abstract

*Reaumuria songarica* is a key dominant species in the desert regions of northern China. The selection of appropriate seed source and the determination of optimal seed harvesting times are critical for the development and utilization of its germplasm resources. In this study, nine *R. songarica* habitats with varying precipitation levels were identified in the HeHuang Valley. We determined the optimal seed harvesting period and evaluated seed traits, germination characteristics, and initial seedling growth at this optimal period harvest time. The results indicated that the optimal harvesting window for *R. songarica* seeds is between 60 and 100 days after flowering, depending on the precipitation of the seed source area. Species origin accounted for the largest variation in seed germination characteristics and seedling growth, with the highest coefficient of variation observed in the vitality index (28.44%) and the lowest in seed viability (3.78%). In the standard germination test, seed germination was primarily influenced by 1000‐seed weight and seed viability, while seedling growth was mainly affected by seed electrical conductivity. Seed drought germination resistance was most influenced by seed biofilm integrity and seed size. In conclusion, high‐quality, drought‐tolerant *R. songarica* germplasm can be obtained by selecting drought‐prone seed source areas in the HeHuang Valley and harvesting seeds between 90 and 100 days after flowering. These findings underscore the critical role of precipitation at the seed source in shaping seed and seedling traits, providing valuable guidance for the future development and utilization of *R. songarica* germplasm resources.

## Graphical abstract

The seeds from nine Reaumuria songarica provenance sites in Chengguide County, Qinghai Province, China were analyzed and studied. The best harvest time and the best provenance were selected.

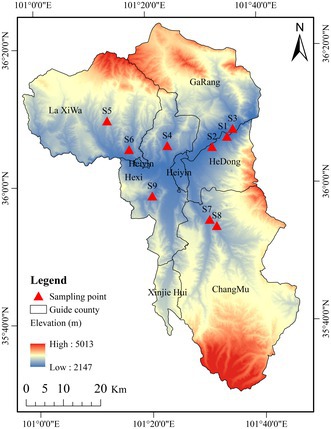

## 1 Introduction

Drought is one of the most pervasive and detrimental abiotic stresses affecting plant growth and development, influencing all stages of the plant life cycle (Ding et al. 2024; Moore et al. 2008; Shah et al. 2017; Zhang et al. 2022). Its impacts include, but are not limited to, inhibited growth, delayed development (Zhu et al. 2023), disruption of the formation of reproductive organs (Moreno et al. 2024), and hindered seed germination and seedling establishment (Liu et al. 2016; Long et al. 2023). Seed germination and seedling establishment mark the beginning of the plant's life cycle, and drought stress during this period can delay germination and weaken seedlings (Long et al. 2023; Zhu et al. 2023), ultimately affecting subsequent growth, development, and population renewal (Matias and Jump 2012; Medina‐Villar et al. 2024). Drought stress impedes germination by reducing water availability, limiting seed expansion, and altering membrane permeability and enzyme activities (Guo et al. 2024). Seed germination is generally divided into three stages: the initial stage, where seeds absorb water to reach a substrate threshold (Allen 2003); the intermediate stage, where metabolic processes are activated for rhizome emergence (Bewley and Black 1985); and the final stage, marked by a significant increase in water uptake and the initiation of radicle emergence (Lewandrowski et al. 2016). Seeds from the same species, but from different seed source locations, have varying water requirements for germination. Seeds can only successfully germinate and develop into healthy seedlings when the surrounding moisture exceeds the critical threshold necessary for germination (Guo et al. 2024).

The effects of seed source location on drought tolerance are primarily mediated through parent plant traits (Luo et al. 2019). Environmental factors such as precipitation, temperature, altitude, geographic coordinates (latitude and longitude), and microenvironment vary between seed source locations and can affect plants in multiple ways (Li et al. 2024). Notably, precipitation plays a decisive role in the drought resistance of plants (Shen et al. 2023). Long‐term survival of the same plant species in environments with significant precipitation differences can result in heterogeneity in the drought resistance of its seeds and progeny, a phenomenon known as drought response plasticity (Moran et al. 2017). Specifically, some arid plants growing in arid and semi‐arid regions have developed efficient water‐use strategies that enable them to better adapt to habitats with varying water availability. Several studies have explored the impact of precipitation on seed drought resistance (Fang et al. 2017; Theresa and Brigitta 2015). For example, Fang et al. (2017) found that *Caragana* seeds from arid regions exhibited a higher germination capacity in dry environments compared to those from humid and semi‐humid regions. This is because moisture is not the primary limiting factor for seed germination in humid and semi‐humid regions, and thus, these seeds are less adapted to arid environments. Similarly, Theresa and Brigitta (2015) studied the drought germination resistance of herbs found above and below the treeline, discovering that herbs below the treeline were significantly more drought‐resistant than those above. This difference results not only from environmental selection but also from plant adaptation to their respective environments.

In addition to this, seeds vary in their ability to resist drought emergence at different stages of development and molding. Seed vigor is a characteristic that determines the potential for rapid and uniform seedling emergence; timely harvesting is a key factor in obtaining highly viable seeds (Gu et al. 2017; Mondo et al. 2013). Yang et al. made a detailed study on the germination ability of *Leymus chinensis* seeds at different harvesting periods and found that timely harvesting could obtain germinative germplasm with strong germination ability, and that either too early or too late harvesting would lead to a reduction in the germination ability of seeds (Yang et al. 2019). There is a gap in such research on ecological grass species, especially dry shrubs growing in arid zones. A great deal of research has been done on the physiological mechanisms corresponding to seed germination and seedling response to drought or osmotic stress, and studies on the entire growing season have been limited to crops such as maize (Gu et al. 2017; Mondo et al. 2013). Using plant species of great ecological value as research subjects can avoid problems such as low seed emergence and seedling survival caused by incorrect harvesting time.

*Reaumuria songarica* is a small, ultra‐arid shrub of the genus *Reaumuria* in the Tamaricaceae family (Lin et al. 1985). It is widely distributed across arid and semi‐arid regions of China, primarily north of the Qinling–Huai River (Wang et al. 2024). *R. songarica* has good palatability and its young leaves are consumed by all major livestock species such as sheep and camels (Liu and Ren 1990; Wang et al. 2025). With exceptional drought tolerance and ecological adaptability, *R. songarica* has become a key species for ecological restoration. However, under natural field conditions, its primary mode of reproduction is asexual (Zhanlin et al. 2012), as the extreme aridity of its habitat often prevents seeds from acquiring sufficient moisture to germinate. It has been shown that dormancy does not exist in *R. songarica* seeds (Guo 2023). Therefore, it can be concluded that the drought‐resistant germination ability of *R. songarica* is mainly controlled by seed source and maturity. This study is important for the effective application of *R. songarica* in desertification restoration efforts.

Despite the ecological value of some plants, little is known about the best place and time to harvest the seeds of these plants. In this study, we investigated the drought germination capacity of *R. songarica* seeds, a small shrub with significant ecological value. In Guide County (Qinghai Province, China), nine experimental locations were established across a gradient of precipitation levels. Seeds were systematically harvested every 10 days post‐anthesis. We proposed the following hypotheses: (i) precipitation differences among seed source areas significantly influence the optimal harvesting period of *R. songarica* seeds; (ii) variations in precipitation at seed source locations result in heterogeneity in seed traits, germination characteristics, drought tolerance, and seedling growth; and (iii) seeds originating from arid regions demonstrate enhanced drought tolerance and superior seedling establishment capacity under water‐limited conditions.

## 2 Materials and Methods

### 2.1 Overview of the Study Area

Guide County in Qinghai Province is located in the HeHuang Valley on the Qinghai‐Tibet Plateau. It encompasses the high, medium, and low suitable areas of *R. songarica* and is an ideal research area for *R. songarica*. Therefore, we chose Guide County as the study area (Wang et al. 2024). The region has a typical plateau continental climate, characterized by distinct seasonal variations, cold winters, warm summers, and significant diurnal temperature fluctuations. The average annual precipitation is 256.8 mm, and the average annual temperature is 8.1°C (Data from China Meteorological Data Network: http://data.cma.cn/). The local vegetation is dominated by drought‐ and salinity‐tolerant shrubs, including *R. songarica*, *Asterothamnus centraliasiaticus*, *Tamarix chinensis* , *Corethrodendron multijugum*, and *Zygophyllum xanthoxylum*, as well as dry herb species such as *Leymus secalinus* and *Oxybasis glauca* (Zhou et al. 2024).

### 2.2 Seed Collection

**FIGURE 1:**
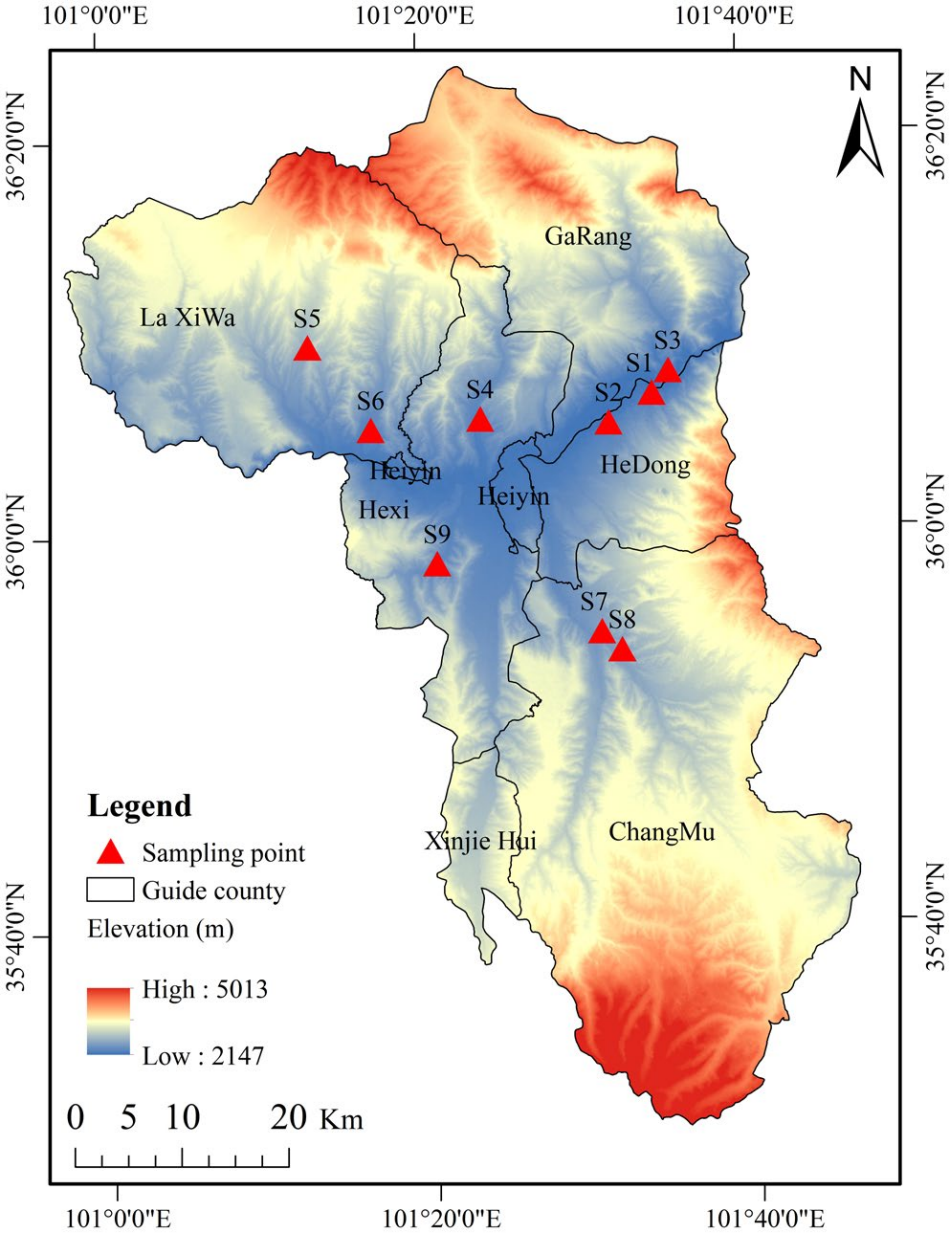
Schematic distribution of the nine *Reaumuria songarica* source locations.

In this study, *R. songarica* seeds were collected from nine seed source locations with varying precipitation levels (Figure 1); detailed information is provided in Table 2. The specific seed collection time is shown in Table S1. Latitude, longitude, and elevation of the source locations were precisely recorded using a handheld global positioning system prior to seed harvesting. Precipitation data for each seed source were obtained from the China Meteorological Data Network (http://data.cma.cn/). Environmental heterogeneity led to variations in flowering times for *R. songarica* across the source locations (Table 2). Seeds were collected at 10‐day intervals after flowering, with three populations within 2 km of each source location randomly selected for repeat sampling. To minimize experimental errors caused by differences in flowering times within populations, seeds exhibiting the best maturity at the time of collection were chosen. A total of 7–10 seed collections were made from each source location, resulting in 76 batches of *R. songarica* seeds.

Seeds were collected, transported to the laboratory, and dried at room temperature. The pericarp and other impurities were removed, and the seeds were then stored in a well‐ventilated cloth bag within a seed storage box (Temperature: 15°C, Humidity: 20%) until the subsequent tests.

### 2.3 Seed Viability and Seed Vitality

Seed viability (SV) was determined using the 2,3,5‐Triphenyl tetrazolium chloride (TTC) test by randomly selecting 100 seeds from each batch and repeating it three times (ISTA 1985). The TTC solution concentration was 0.5%, and the staining temperature and time were 30°C for 30 min. Seeds with viability in this study were embryo‐completely or mostly stained seeds. SV was calculated according to the formula (1):

(1) $$\mathrm{SV}\;\left(\%\right)=\frac{\text{Number of colored seeds}}{\text{Number of seeds for testing}}\times 100\%$$

Seed vitality refers to the combination of various potential properties of seeds, including field performance and storage characteristics (Hampton and TeKrony 1995). Since seed vitality cannot be measured directly, this study aimed to characterize seed vitality using the electrical conductivity of the seeds (Ma et al. 2020). For each batch of *R. songarica* seeds, 0.5 g was accurately weighed, and the test was repeated three times. The seeds were rinsed with deionized water and placed in 200 mL wide‐mouth conical flasks containing 100 mL of deionized water. The conical flasks were sealed and incubated at a constant temperature of 25°C for 24 h. The conductivity of the seed leachate was measured using a Ray‐Mag‐307A conductivity meter. The samples were then subjected to a boiling water bath for 15 min, after which the conductivity was measured again once the seed leachate had cooled to room temperature. A blank control, consisting of conical flasks with only 100 mL of deionized water, was used for comparison. Relative conductivity (RC) and absolute conductivity (AC) were calculated using Equations (2) and (3):

(2) $$\mathrm{RC}\;\left(\%\right)=\frac{C_{1}–C_{0}}{C_{2}}$$

(3) $$\mathrm{AC}\;\left(\mathrm{\mu S}\;{\mathrm{cm}}^{-1}\;g^{-1}\right)=\frac{C_{1}–C_{0}}{w}$$

where *C*_1_ is the conductivity of the sample; *C*_0_ is the conductivity of the blank control; *C*_2_ is the conductivity of the seeds after the boiling water bath.

### 2.4 Seed Morphology

In this study, seed size was expressed in terms of the seed aspect ratio and 1000‐seed weight (TSW). Twenty seeds were randomly selected from each batch, observed, and photographed using a Nikon SMZ18 microscope (Model P2‐FIRL). To ensure accurate measurement of SL and SW, the surface of the seeds was briefly moistened with deionized water before photographing, which helped exclude interference from downy hairs without affecting the seed size. SL and SW were measured and analyzed using NIS‐Elements D (version 4.40) software. TSW was determined by weighing 1000 seeds on an analytical balance (accuracy: 0.0001 g), with the measurement repeated three times for each batch. The formula for calculating the seed length‐to‐width ratio (LWR) is as follows:

(4) $$\mathrm{LWR}=\frac{\mathrm{SL}}{\mathrm{SW}}$$

### 2.5 Seed Germination Stress Drought Test

*R. songarica* is a typical drought‐tolerant shrub found in arid and semi‐arid regions. However, drought stress significantly affects its seed germination and early seedling growth. Therefore, this study aimed to investigate the drought tolerance of *R. songarica* seeds sourced from different locations and maturity levels. Specifically, we sought to identify the optimal seed harvesting period and examine the influence of precipitation in the seed source area on seed germination characteristics (SGC), seedling growth, and drought resistance.

This experiment used different concentrations of polyethylene glycol (PEG‐6000) solution to simulate seven drought stress levels: 0 MPa (D0), −0.3 MPa (D1), −0.6 MPa (D2), −0.9 MPa (D3), −1.2 MPa (D4), −1.5 MPa (D5), and −1.8 MPa (D6), with D0 serving as the control (Michel and Kaufmann 1973). Seed germination experiments were conducted in December 2023.

Fifty seeds were randomly selected from each batch, and the procedure was repeated three times. The seed surfaces were first rinsed with distilled water, sterilized with a 5% NaClO solution for 5 min, rinsed again with distilled water for 2 min, and then blotted dry with filter paper. The seeds were evenly placed in 9‐cm diameter sterile Petri dishes containing filter paper, and 7 mL of the corresponding solution was added to each dish. The Petri dishes were incubated in a thermostatic chamber with a 14‐h light period at 25°C, a 10‐h dark period at 20°C, and a relative humidity of 60% (Guo 2023). The solution and filter paper were renewed daily to maintain a constant stress level. Seed germination was recorded every 24 h. Germination was considered complete when the radicle emerged by at least 2 mm, and the experiment ended when no new germination was observed for five consecutive days, for a total duration of 14 days (ISTA 1985). At the end of the experiment, plumule length (PL), radicle length (RL), and seedling fresh weight (FW) were measured, with 10 seedlings per dish; if fewer than 10 seedlings germinated, all were measured.

The following indices were used to assess seed germination and seedling growth: germination rate (GR), germination index (GI), vitality index (VI), germination synchrony index (GSI) (Al‐Mudaris 1998; Maleki et al. 2023), mean germination time (MGT) (Ranal and Santana 2006), germination uniformity (GU) (Al‐Mudaris 1998; Ranal and Santana 2006), germination drought tolerance index (GDTI) (Li et al. 2024), FW, root‐to‐shoot ratio (RSR), and seedling length. The respective formulae were calculated as follows:

(5) $$\mathrm{GR}\;\left(\%\right)=\frac{N}{50}\times 100\%$$

(6) $$\mathrm{GI}=\sum \frac{G_{t}}{D_{t}}$$

(7) $$\mathrm{VI}=\mathrm{GI}\times \left(\mathrm{PL}+\mathrm{RL}\right)$$

(8) $$\mathrm{GSI}=\frac{\sum \left(\frac{G_{t}\left(G_{t}-1\right)}{2}\right)}{\frac{N\left(N–1\right)}{2}}$$

(9) $$\mathrm{MGT}=\frac{\sum \left(G_{t}\times D_{t}\right)}{N}$$

(10) $$\mathrm{GU}=\frac{\mathrm{MGT}-\mathrm{MGT}75}{\mathrm{MGT}75}$$

(11) $$T_{\mathrm{Ri}}=1+|D_{i}|$$

(12) $$\text{GDTI}=\frac{\sum {\mathrm{GI}}_{i}\times T_{\mathrm{Ri}}}{7}$$

(13) $$\mathrm{RSR}=\frac{\mathrm{RL}}{\mathrm{PL}}$$

where *N* is the total number of germinated seeds, *G*_*t*_ is the number of germinated seeds on day *t*, *D*_*t*_ is the corresponding number of days to germination, PL is plumule length, RL is radicle length, MGT75 is the average germination time of 75% of the seeds, and Tri is the weighted assignment coefficient of the drought stress treatments (*T*_Ri_ = 1) *D*_*i*_ is the concentration value of each treatment.

### 2.6 Data Analysis

All data were compiled and summarized using Microsoft Excel 2019 and analyzed by one‐way ANOVA in SPSS 20 with a significance level of *p* < 0.05. Data presented in the graphs and tables are reported as mean ± standard error. All ratio data (e.g., GR, SV) were transformed using the square root inverse chord method. In this study, six indicators such as GDTI, GR, GI, MGT, GSI, and GU were used to characterize seed germination characterization; five indicators such as FW, PL, RL, RSR, and VI were used to characterize seedling growth status. The technique for order preference by similarity to an ideal solution (TOPSIS) was employed to comprehensively evaluate the optimal harvesting time of *R. songarica* across seed source locations, with the harvest time closest to the ideal solution identified as the optimal decision (Yoon and Hwang 1995). A mixed‐effects model was established using the “lmer” function in the lmerTest package in R 4.3.2, with LWR, SL, SW, seed TSW, RC, AC, and SV as fixed effects and populations treated as random effects. The Shapiro–Wilk test was performed to verify the normality of all variables prior to statistical analyses. A piecewise structural equation model was constructed using the PiecewiseSEM package in R 4.3.2 to explore the effects of precipitation at seed sources on GDTI, seed germination characteristics (SGC), and seedling growth state (SGS), as well as the associated influence processes and path coefficients; principal component analysis was conducted using the “psych” package in R 4.3.2, and the first principal component was extracted as SGC and SGS. Among them, SGC was obtained by dimensionality reduction of GDTI, GR, GI, MGT, GSI, and GU, and SGS was obtained by dimensionality reduction of FW, PL, RL, RSR, and VI. The full names and units of all abbreviations in this study are listed in Table 1. Plots were generated using Origin 2021.

**TABLE 1:** Traits measured in this study with abbreviations, full titles, and units.

Abbreviation	Full title	Units
CV	Coefficient of variation	%
VI	Vitality index	—
TSW	1000‐seed weight	g
SW	Seed width	mm
SV	Seed viability	—
SL	Seed length	mm
SGS	Seedling growth state	—
SGC	Seed germination characteristics	—
RSR	Root‐to‐shoot ratio	—
RL	Radicle length	mm
RC	Relative conductivity	%
PRE	Annual precipitation	mm
PL	Plumule length	mm
MGT	Mean germination time	d
LWR	Seed length‐to‐width ratio	—
GU	Germination uniformity	—
GSI	Germination synchrony index	—
GR	Germination rate	—
GI	Germination index	%
GDTI	Germination drought tolerance index	—
FW	Seedling fresh weight	g
AC	Absolute conductivity	μS cm^−1^·g^−1^

**TABLE 2:** Detailed information on the nine *R. songarica* source locations.

Sampling point	Longitude and latitude	Elevation	Annual precipitation (2022.5–2023.4)	Date of flowering (year‐month‐day)	The end date of flowering (year‐month‐day)	Sampling times
S1	101°34′16.24″ E 36°06′51.87″ N	2248 m	205.68 mm	2023‐7‐6	2023‐8‐20	10
S2	101°31′33.37″ E 36°05′25.19″ N	2197 m	224.43 mm	2023‐7‐6	2023‐8‐20	10
S3	101°35′26.24″ E 36°07′57.02″ N	2335 m	211.79 mm	2023‐7‐6	2023‐8‐20	10
S4	101°23′33.62″ E 36°05′43.65″ N	2319 m	306.68 mm	2023‐7‐29	2023‐9‐5	8
S5	101°12′54.09″ E 36°09′31.20″ N	2701 m	346.68 mm	2023‐8‐10	2023‐9‐15	7
S6	101°16′42.97″ E 36°05′16.90″ N	2337 m	329.99 mm	2023‐7‐29	2023‐9‐5	8
S7	101°30′49.37″ E 35°54′51.56″ N	2532 m	440.16 mm	2023‐7‐29	2023‐9‐5	8
S8	101°32′02.93″ E 35°53′55.79″ N	2584 m	417.60 mm	2023‐7‐29	2023‐9‐5	8
S9	101°20′39.97″ E 35°58′27.57″ N	2400 m	457.19 mm	2023‐8‐10	2023‐9‐15	7

**TABLE 3:** TOPSIS comprehensive evaluation was used to analyze the optimum harvest period of various sources.

Sampling point	TOPSIS	Seed collection time									
		T1	T2	T3	T4	T5	T6	T7	T8	T9	T10
S1	Ci	0.33	0.22	0.35	0.48	0.49	0.63	0.65	0.64	0.63	0.67
	Rank	9	10	8	7	6	5	2	3	4	1
S2	Ci	0.31	0.19	0.42	0.5	0.56	0.65	0.69	0.62	0.69	0.66
	Rank	9	10	8	7	6	4	2	5	1	3
S3	Ci	0.32	0.21	0.38	0.5	0.54	0.67	0.67	0.65	0.66	0.69
	Rank	9	10	8	7	6	3	2	5	4	1
S4	Ci	0.17	0.21	0.52	0.73	0.79	0.83	0.66	0.77	—	—
	Rank	8	7	6	4	2	1	5	3	—	—
S5	Ci	0.48	0.6	0.59	0.68	0.61	0.7	0.69	—	—	—
	Rank	7	5	6	3	4	1	2	—	—	—
S6	Ci	0.39	0.35	0.5	0.63	0.65	0.67	0.63	0.63	—	—
	Rank	7	8	6	4	2	1	3	5	—	—
S7	Ci	0.19	0.38	0.28	0.6	0.68	0.81	0.7	0.79	—	—
	Rank	8	6	7	5	4	1	3	2	—	—
S8	Ci	0.31	0.55	0.62	0.64	0.64	0.71	0.67	0.77	—	—
	Rank	8	7	6	4	5	2	3	1	—	—
S9	Ci	0.33	0.43	0.64	0.71	0.68	0.66	0.73	—	—	—
	Rank	7	6	5	2	3	4	1	—	—	—

**TABLE 4:** A study of the coefficient of variation of seed characteristics of *R. songarica*.

Group	Character	CV %	Max/Min
Seed characterization	SV	3.7812	1.1101
	RC	8.3147	1.2409
	AC	9.6745	1.3434
	TSW	8.0509	1.2331
	SL	4.0809	1.1390
	SW	8.6842	1.2632
	LWR	9.2186	1.2909
Seed germination characterization	GDTI	18.2541	1.9257
	GR	5.3008	1.1679
	GI	14.0662	1.5160
	MGT	13.9505	1.5521
	GSI	13.2064	1.4880
	GU	11.6457	1.4567
Seedling growth status	FW	19.6866	1.7684
	PL	9.5450	1.2949
	RL	23.8811	1.9873
	RSR	16.4777	1.6028
	VI	28.4395	2.6361

*Note:* The seed characterization group indices: seed viability (SV), relative conductivity (RC), absolute conductivity (AC), 1000‐seed weight (TSW), seed length (SL), seed width (SW), and seed length‐to‐width ratio (LWR). The seed germination characterization group indices: germination drought tolerance index (GDTI), germination rate (GR), germination index (GI), mean germination time (MGT), germination synchrony index (GSI), and germination uniformity (GU). The seedling growth status group indicators: seedling fresh weight (FW), germ length (PL), root length (RL), root‐to‐crown ratio (RSR), and vigor index (VI). The full names and units of all abbreviations in this study are listed in Table 1.

## 3 Results

### 3.1 Screening for the Optimal Harvesting Period of *R. songarica* Seeds

Based on the intrinsic correlations among the indicators, PL, RL, SV, MGT, GSI, GU, GDTI, AC, LWR, and TSW were incorporated into the TOPSIS comprehensive evaluation system to determine the optimal seed harvesting periods for *R. songarica* across the nine seed source locations. The results, presented in Table 3, indicate that the optimal harvesting periods for seed source locations S1 to S9 are T10, T9, T10, T6, T6, T6, T6, T8, and T7, respectively. Analysis of the precipitation conditions at the various source locations revealed that in arid areas with lower precipitation (S1, S2, and S3), *R. songarica* exhibited longer intervals between flowering and seed harvesting, suggesting an extended period for nutrient transfer to the seeds. Subsequent studies will focus on the nine batches of *R. songarica* seeds identified in this section.

### 3.2 Variability of Seed Traits

Analysis of variance (ANOVA) revealed significant differences (*p* < 0.05) in germination characteristics and seedling growth status among the nine *R. songarica* germplasm. In the seed trait group, only AC and TSW were statistically significant (Table 4). Overall, the variation magnitude was highest in the seedling growth status group, followed by the SGC group, and then the seed characterization group. Among seed traits, AC exhibited the largest coefficient of variation (CV) at 9.67%, while SV showed the smallest CV at 3.78%. These findings suggest that differences in seed source location significantly affected the development of the plasma membrane system in *R. songarica* seeds but had minimal impact on SV and GR (Table 4).

**FIGURE 2:**
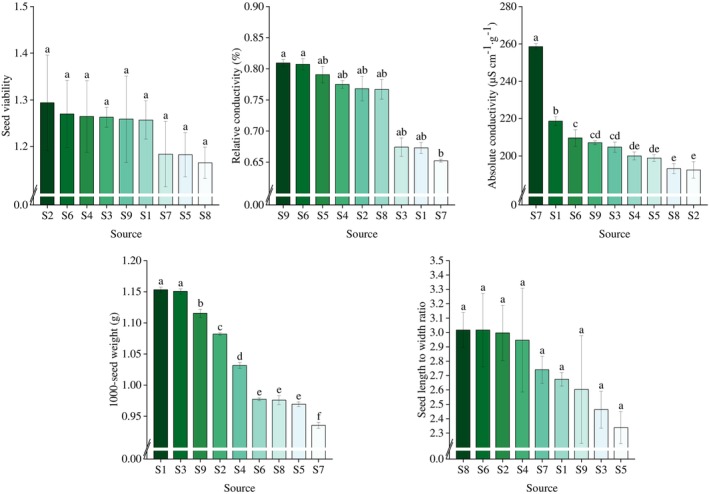
Variation in seed characterization parameters of *R. songarica* in various source locations. Different lowercase letters indicate significant differences, unless otherwise stated.

A one‐way ANOVA was performed to analyze the parameters characterizing *R. songarica* seeds at the optimal harvesting period across the nine seed source locations (Figure 2). For SV, no significant differences were observed among the source locations, suggesting that *R. songarica* seeds that successfully completed their developmental cycle exhibited high viability. This may indicate a higher potential for germination under ideal conditions. The AC of seeds from locations S2 and S8 was significantly lower than that of seeds from other locations, implying that the plasma membrane system of *R. songarica* seeds from these locations was more developed, with better cell integrity and higher SV, leading to enhanced seedling emergence under challenging location conditions. Seed LWR showed no significant differences among the source locations, indicating that seed source locations did not affect the maturity or fullness of *R. songarica* seeds. However, TSW was significantly higher in seeds from source locations S1 and S3 compared to the other locations.

### 3.3 Variability of Seed Germination Characteristics

The CV results (Table 4) revealed significant variations in the SGC group, including GDTI, GI, MGT, GSI, and GU, with coefficients of 18.25%, 14.07%, 13.95%, 13.21%, and 11.65%, respectively. These variations suggest considerable differences in the speed of germination, uniformity of germination, and drought tolerance among *R. songarica* seeds from different source locations. However, the CV for GR was only 5.30%; confirming our earlier hypothesis that the viability of *R. songarica* seeds would not vary significantly across different seed source locations, provided the seeds underwent a full reproductive cycle. Furthermore, this suggests that the GR remains relatively stable under ideal conditions.

**FIGURE 3:**
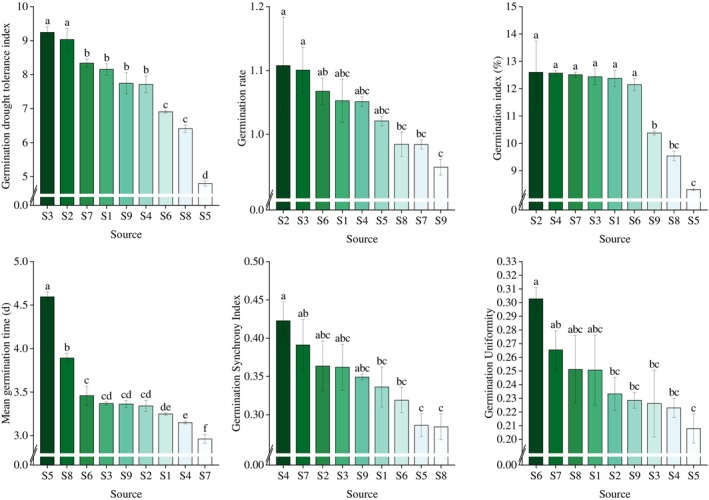
Variation in characteristic parameters of *R. songarica* seed germination in various source locations.

The seed germination test results revealed significant differences in germination parameters, including GDTI, GR, GI, MGT, GSI, and GU, among *R. songarica* seeds from different source locations (Figure 3). The GDTI for seed sources S2 and S3 was significantly higher than that for other sources, indicating superior drought tolerance. GR was significantly higher in sources S2 and S3 compared to S7, S8, and S9. After arcsine square root transformation, GR values for all sources remained within the range of 0.95 to 1.11, showing small variations despite the differences; GR of other batches at each location is shown in Table S2. In the seed germination characterization group, GI, MGT, and GSI, which reflect seed germination speed, indicated that the germination rates of sources S2, S4, and S7 were relatively fast (Figure 3). GU, a measure of germination uniformity, was highest for seeds from sources S3, S4, and S5, suggesting better uniformity in these locations (Figure 3). In conclusion, seeds from seed source locations S2, S3, and S4 demonstrated uniform germination within a concentrated time frame.

### 3.4 Variability of Seedling Growth State

**FIGURE 4:**
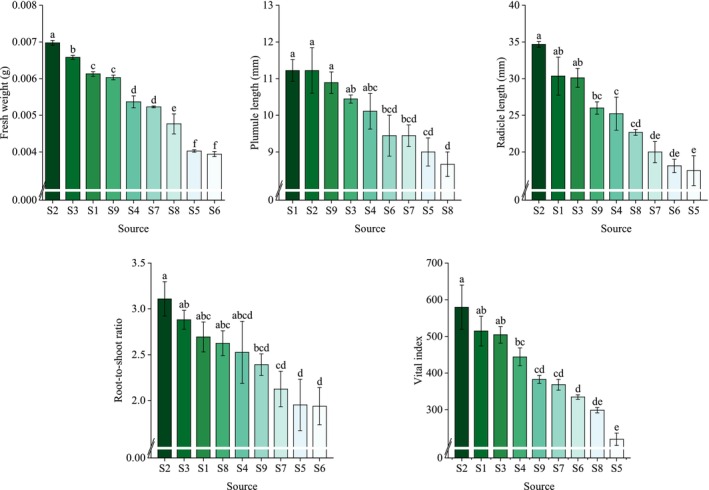
Changes in characteristic parameters of *R. songarica* seedling growth in various source locations.

The CV analysis revealed that the variation in the growth state parameters of *R. songarica* seedlings was consistently above 9.55%. Among these parameters, the VI exhibited the largest CV (28.44%), followed by RL at 23.88%. This high variation in RL may be attributed to its larger range, spanning from 17.45 to 34.67 mm (Figure 4). In contrast, the CV for PL was the smallest (9.55%), likely due to its narrower range, from 8.67 to 11.22 mm (Figure 4).

At the conclusion of the germination test, seedling FW, PL, and RL of *R. songarica* seedlings were measured. A one‐way ANOVA revealed significant variation in seedling growth status among different seed source locations. FW, PL, and RL were significantly higher at source location S2 compared to the other locations, suggesting that *R. songarica* seedlings from S2 were larger and had greater developmental potential. Additionally, RSR of seedlings from S1, S2, and S3 was higher than that of seedlings from other locations. This indicates that seedlings from these three locations had a larger root proportion, which likely enhanced their ability to absorb water and nutrients, resulting in better survival under extreme environmental conditions. The results from the VI further supported this conclusion.

### 3.5 Seed Germination and Seedling Growth in Relation to Seed Characteristics

**FIGURE 5:**
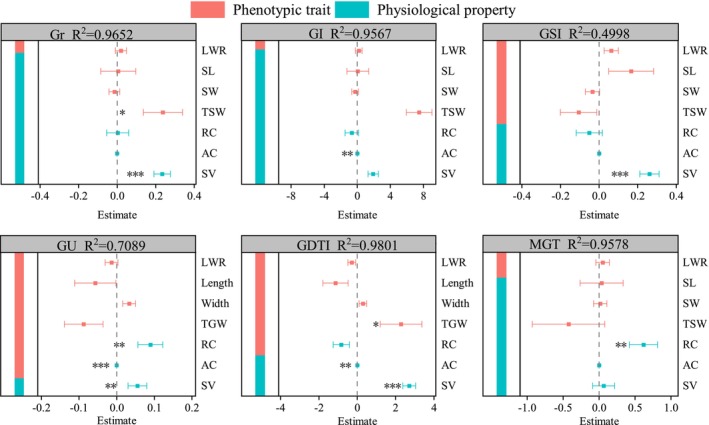
Influence of seed characteristics on seed germination characteristics (SGC) of *R. songarica* from nine seed source locations. Note: The * indicates a significant difference at the 0.05 level. ** indicates a significant difference at the 0.01 level; *** indicates a significant difference at the 0.001 level.

Seed trait indicators were categorized into two groups: phenotypic traits, including seed LWR, SL, SW, and TSW; and physiological properties, including RC, AC, and SV. The mixed‐effects model revealed a significant relationship between *R. songarica* SGC and seed traits (Figure 5). Decomposition of the model showed that seed traits explained 96.52%, 95.67%, 95.78%, 49.98%, 70.89%, 98.01%, and 95.78% of the variation in GR, GI, GSI, GU, GDTI, and MGT, respectively. Among these, phenotypic traits had a greater influence on GSI, GU, and GDTI, while physiological properties had a stronger effect on GR, GI, and MGT.

The variables influencing seed germination parameters of *R. songarica* varied across the nine seed source locations (Figure 5). SV and TSW significantly influenced GR, while AC and RC had a significant effect on GI and MGT, respectively. These results suggest that the integrity of the plasma membrane plays a crucial role in determining the speed of germination. Additionally, SV, AC, and TSW were identified as key factors affecting the drought‐resistant germination ability of the seeds.

**FIGURE 6:**
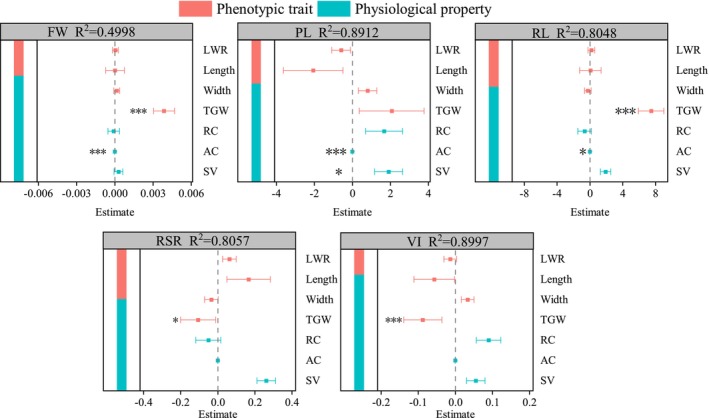
Influence of seed characteristics on seedling growth state (SGS) of *R. songarica* from nine seed source locations. Note: The * indicates a significant difference at the 0.05 level. ** indicates a significant difference at the 0.01 level; *** indicates a significant difference at the 0.001 level.

A significant relationship was also observed between seedling and seed traits in *R. songarica* (Figure 6). Effect decomposition analysis revealed that seed traits explained 49.98%, 89.12%, 80.48%, 80.57%, and 89.97% of the variation in seedling FW, PL, RL, RSR, and VI, respectively. Seed physiological properties had a greater influence on seedling growth status. TSW was the only seed trait that significantly affected RSR and VI; it also had a significant impact on FW and RL. These findings suggest that TSW significantly influences FW and root development, thus playing a key role in the subsequent growth potential of seedlings. Additionally, seedling size parameters (FW, PL, RL) were significantly affected by AC, indicating that the integrity of the seed's biofilm is essential for the healthy growth of *R. songarica* seedlings.

### 3.6 The Influence of Drought on Seed Germination and Seedling Growth

**FIGURE 7:**
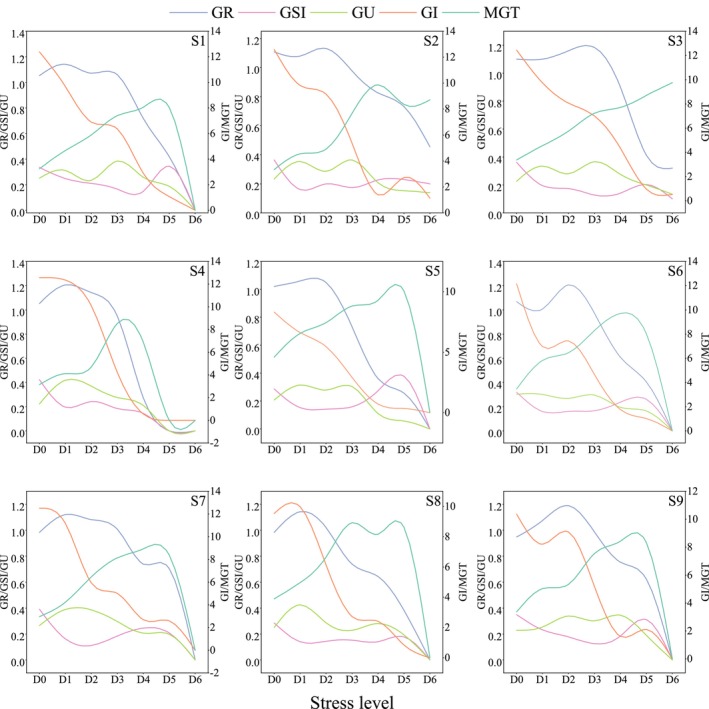
Characteristics of variation in drought‐resistant germination parameters of *R. songarica* seeds from various source locations.

**FIGURE 8:**
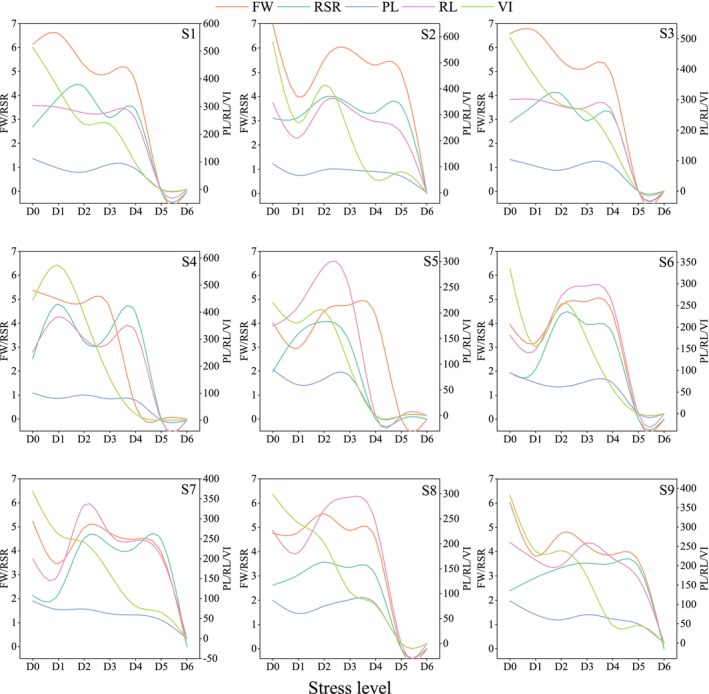
Characteristics of variation in drought‐resistant growth parameters of *R. songarica* seedlings invarious source locations.

Drought stress germination experiments on *R. songarica* seeds from various source locations at the optimal harvesting period were conducted over 14 days. By monitoring germination parameters under different water potential gradients, the results were presented in Figure 7. Similar to the standard germination test, seedling FW, as well as PL and RL, were measured at the end of the experiment to assess seedling growth status (Figure 8).

Figure 7 shows that as environmental water potential decreases, the GR of *R. songarica* seeds from different source locations initially increases slightly before declining sharply. GI generally decreases, with the decline becoming more pronounced as water potential decreases. However, the reduction in GU is minimal. These results suggest that mild water stress may promote *R. songarica* seed germination to some extent, but more severe declines in water availability strongly inhibit germination rates, with less impact on overall germination uniformity. Except for the seeds from source locations S2 and S3, MGT for all other source locations drops to zero after the D5 treatment. This indicates that at the D6 stress level, seeds from these locations fail to germinate, reaching the drought germination threshold.

Figure 8 illustrates that the FW of *R. songarica* seedlings decreased under drought stress. Notably, while the GR of seeds from source locations S2 and S3 at the D6 stress level was not zero, their FW dropped to zero. This indicates that seeds from these locations met the germination criterion (radicle protrusion of 2 mm through the seed coat) but failed to develop into complete seedlings under D6 stress, suggesting that this level of drought stress is approaching the critical threshold for these sources. The trend of PL was similar to that of GU. Since PL is relatively short, the decrease caused by drought stress was limited, and seeds failed to develop into complete seedlings once drought stress surpassed the threshold. In contrast, RL exhibited a significant downward trend with marked decline points, occurring at D3, D3, D4, D4, D2, D3, D2, D3, and D3 across seed sources S1 to S9, respectively. RSR of *R. songarica* seedlings initially increased under moderate water stress, indicating enhanced root development for resource uptake, but declined under severe stress, reflecting growth limitations. A sharp decline in VI underscores the substantial threat posed by drought stress to the growth of *R. songarica* seedlings, with the severity of this threat intensifying as stress levels increase.

### 3.7 Processes of Precipitation at Source of Seed on GDTI, SGC, and SGS

**FIGURE 9:**
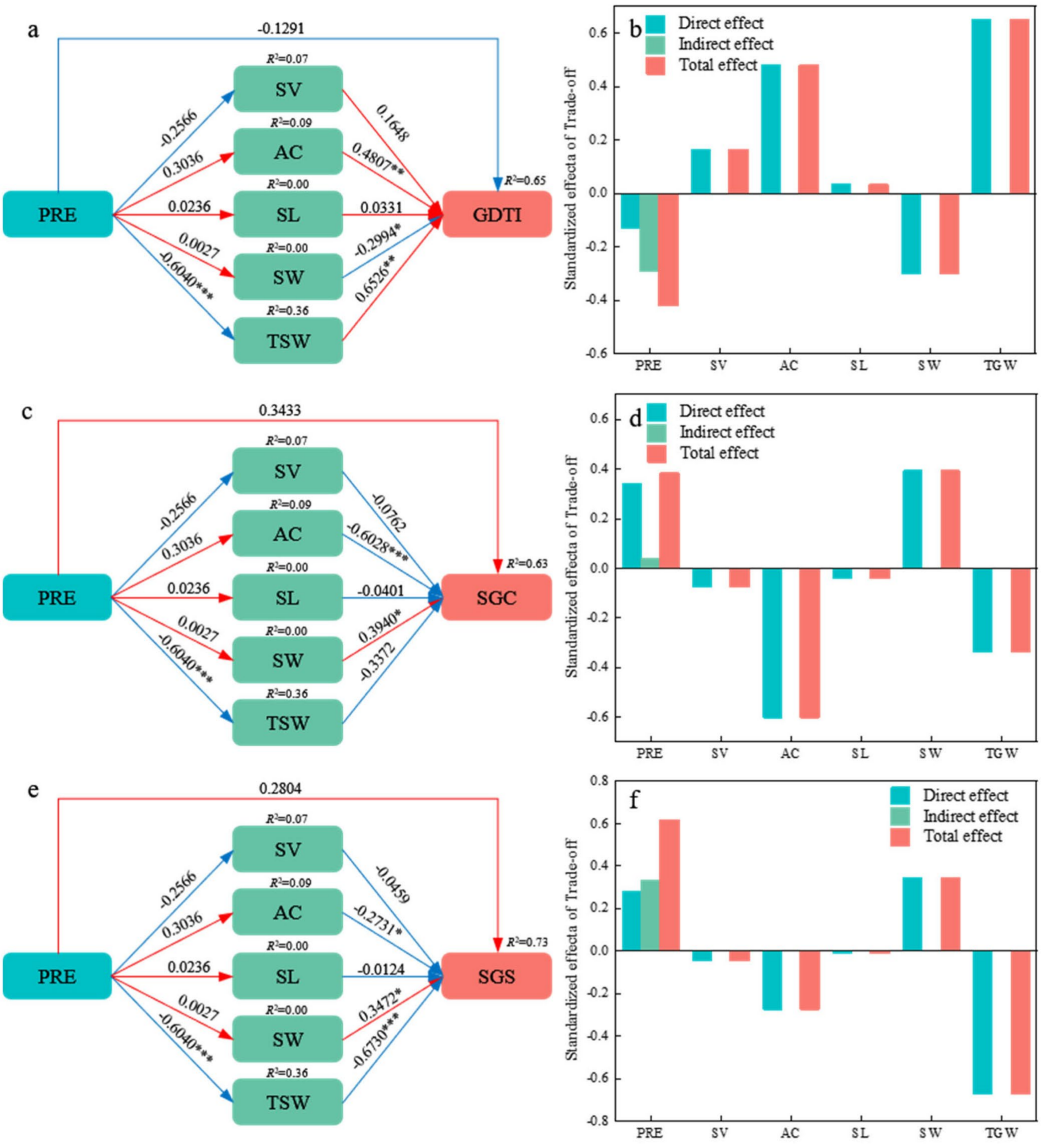
Structural equation model analysis of the influence path of PRE on GDTI (a), SGC (c), SGS (e) of *R. songarica* seeds, and standardized effect values of each variable (b, d, f). The red and blue arrows represent positive and negative path relationships, respectively. The values are standardized path coefficients, and the variance explained by the model is expressed as *R* ^2^. Precipitation at the source of seed (PRE). *: *p* < 0.05; **: *p* < 0.01; ***: *p* < 0.001.

A piecewise structural equation model was employed to investigate how PRE influenced SV, AC, SL, SW, TSW, and their subsequent effects on GDTI (Figure 9a), SGC (Figure 9c), and seedling growth state (SGS) (Figure 9e). According to the research results of the mixed‐effects model, SV, AC, SL, SW, and TSW were included in the piecewise structural equation model. The model demonstrated a good fit (*Fisher's C* = 10.744, *p* = 0.953). Collectively, PRE, SV, AC, SL, SW, and TSW accounted for 65%, 63%, and 73% of the variation in GDTI, SGC, and SGS, respectively, for *R. songarica* seeds from the nine sources.

PRE had a significant direct effect on TSW of *R. songarica* seeds (*p* < 0.05), with a path coefficient of −0.6040 (Figure 9a,c,e). AC, SW, and TSW significantly influenced GDTI of *R. songarica* seeds, with path coefficients of 0.4807, −0.2994, and 0.6526, respectively (Figure 9a). AC and SW had direct significant effects on SGC, with path coefficients of −0.6028 and 0.3940 (Figure 9c). Similarly, AC, SW, and TSW significantly affected SGS, with path coefficients of −0.2731, 0.3472, and −0.6730, respectively (Figure 9e). The total effects, calculated as the sum of direct and indirect effects, indicated that PRE influenced GDTI and SGS primarily through its effect on TSW, with total effects of 0.6526 and −0.6730, respectively (Figure 9b, Figure 9f). Additionally, PRE affected SGC mainly through its impact on AC, with a total effect of −0.6028 (Figure 9d).

## 4 Discussion

### 4.1 Screening for Optimal Harvesting Period

Timely harvesting of seeds once they reach developmental maturity is crucial for obtaining healthy, high‐quality seeds and preventing degradation due to improper timing (Finch‐Savage and Bassel 2015). For instance, the optimal harvesting period for different *L. chinensis* germplasms varies, and harvesting seeds too early or too late can result in suboptimal germination rates (Yang et al. 2019). In our study, the optimal harvesting period for *R. songarica* germplasm differed by up to 40 days across nine distinct seed source locations, influenced by precipitation (Table 3). We attribute this variation primarily to differences in the flowering time of *R. songarica* across seed source locations, which is driven by variations in precipitation. According to S.C. Stearns' theory of the trade‐off between survival and reproduction, plants in water‐limited environments are constrained in the time available for survival. If too many resources are allocated to vegetative growth, reproductive growth may fail to occur in time, compromising the plant's ability to reproduce. As a result, plants accelerate their reproductive growth to ensure species continuation. This strategy, shaped by natural selection, favors individuals capable of completing their life cycle quickly in water‐limited environments (Stearns 1992). However, this reproductive strategy can be detrimental when precipitation increases, as it may lead to excessive vegetative growth in *R. songarica*, limiting reproductive growth time, reducing nutrient transfer to seeds, and ultimately decreasing seed quality (Mactavish and Anderson 2020). This inference is supported by subsequent studies on seed germination and seedling growth. In summary, our findings suggest that moderately arid seed source locations are associated with prolonged reproductive growth and higher seed quality for *R. songarica*. This is consistent with the characterization of *R. songarica* as an arid‐adapted species. Future studies using seeds from various source locations harvested at optimal times will provide a clearer demonstration of how source location affects *R. songarica* seed production. These results support our initial hypothesis regarding the optimal harvesting period for *R. songarica* seeds.

### 4.2 Seed Traits, Germination, and Seedling Growth in Response to Seed Source Location

When environmental conditions change, each plant organ exhibits specific adaptive responses (Thorogood et al. 2018). However, in the present study, we observed minimal variation in the seed traits of *R. songarica* from different seed source locations. This may be attributed to the fact that we selected seeds from the optimal harvesting period at each location, ensuring that the seeds had completed their developmental stages, thereby minimizing variation in basic seed traits (Li et al. 2024). Additionally, seed phenotypic traits are among the least plastic of all plant characteristics (Vilela and Gonzalez‐Paleo 2015). Among them, the CV of *R. songarica* SV was the smallest with only 3.7812% in various source locations (Table 4) and there was no significant difference between the SVs of various source locations (Figure 2), which suggests that a complete reproductive period ensures that *R. songarica* obtains a high SV and has a high potential for germination in an ideal environment. Nevertheless, significant differences were observed in AC and TSW (Table 4), suggesting that seed source location can influence seed size and vigor. Elevated AC indicates higher electrolyte leakage within the seed cells, which impairs the seed's ability to germinate and develop into a seedling under challenging environmental conditions (Ma et al. 2020). Significant differences in TSW further suggest that *R. songarica* seeds from different sources vary in their nutrient retention capacity, with larger TSW seeds providing better support for progeny seedlings facing extreme environments (Amusa et al. 2020). This suggests that changes in precipitation at the seed source location have limited ability to drive variation in seed phenotypic traits but affect seed cell membrane integrity as well as nutrient storage capacity. Similarly, in extreme environments, plants tend to retain seeds with a higher likelihood of survival (Wu et al. 2022).

The germination ability of *R. songarica* seeds is primarily influenced by the seed source location. Variations in germination performance under optimal conditions are driven by two main factors: genetic differentiation (Linhart and Grant 1996; Thorogood et al. 2018) and seed quality, which is influenced by the amount of nutrients transferred from the mother plant to the next generation (Falleri 1994). The latter is primarily shaped by environmental factors. In this study, *R. songarica* seeds exhibited significant variation in germination traits, such as GDTI, germination speed, and germination uniformity, especially between high‐ and low‐precipitation seed source locations (Figure 3). Specifically, GDTI was significantly higher in seed source locations S2 and S3 compared to the other locations, and seeds from low‐precipitation locations germinated more quickly, indicating the inheritance of drought tolerance from the parent plants. Since *R. songarica* predominantly inhabits arid regions with minimal rainfall (Wang et al. 2024), it has evolved enhanced resource utilization capabilities, enabling rapid germination when suitable conditions arise. However, GR did not exhibit significant variation across the germplasm gradient, likely due to minimal differences in SV. Each batch of seeds was classified into viable and nonviable categories (Jacob et al. 2022), with viable seeds further categorized into dormant and nondormant groups (Finch‐Savage and Bassel 2015). Seeds that are capable of germinating under optimal conditions must be both viable and nondormant (Gao 2020).

Seedling growth is influenced by both the growing environment and seed quality. Under consistent growth conditions, variations in seedling growth are primarily attributed to differences in seed quality. In this study, *R. songarica* seedlings from nine different seed source locations exhibited significant variation in FW, RL, and VI (Table 4). Overall, seedlings from arid source locations were larger and exhibited higher potential for future growth. This phenomenon may be attributed to the extended reproductive growth period in arid zones, enabling *R. songarica* to allocate more nutrients to its offspring, enhancing their ability to withstand environmental extremes. These findings support our second hypothesis.

### 4.3 Influence of Seed Traits on Germination Characteristics and Seedling Growth Status

SGC are primarily influenced by seed traits. In this study, GR was significantly affected by TSW and SV; germination speed by seed electrolyte conductivity; and germination uniformity by AC, RC, and SV. Additionally, germination duration (GDTI) was significantly influenced by TSW, AC, and SV (Figure 5). TSW serves as an indicator of the nutrient content in seeds (Amusa et al. 2020), and these findings suggest that nutrient storage plays a crucial role in both seed germination and drought resistance (Li et al. 2024). The selective permeability of seed biofilms influences various germination traits, including germination speed and uniformity. In this study, phenotypic traits were primarily associated with nutrient storage in *R. songarica* seeds, whereas physiological traits were more related to the internal structure and integrity of the seed biofilm. Therefore, we conjecture that the internal structure and biofilm integrity of *R. songarica* seeds govern seed germination performance and drought resistance.

Building on previous research, we believe that seed traits influence the growth of subsequent seedlings (Jaybhaye et al. 2024; Kennedy et al. 2004; Sousa and Mitchell 2003). In this study, physiological characteristics had a greater impact on the growth of *R. songarica* seedlings. During germination, seeds utilize their stored nutrients solely for the germination process. Once germination is complete, seedling growth depends on photosynthesis for energy production and nutrients absorbed by the root system (Lloret et al. 1999; Wulff 1986). Our findings indicate that seedling growth varies across seed source locations, and we hypothesize that this variation is primarily driven by the integrity of the seed's internal structure and the development of its cellular biofilm, suggesting a strong positive correlation. This result supports our final hypothesis.

We hypothesize that the differences in seed source locations in this study are primarily driven by variations in precipitation, which influence the domestication of seed traits. These variations, in turn, affect SGC and subsequent seedling growth.

### 4.4 Seed Drought Adaptability of *R. songarica* Driven by Provenance

As an arid plant, *R. songarica* exhibits a high tolerance to drought and has developed several adaptations to cope with water scarcity (Li et al. 2018; Xie et al. 2020). Seed germination is enhanced under low drought stress conditions, which explains why a low concentration of PEG solution has been used as a seed priming agent in some studies (Jisha et al. 2013). The drought tolerance thresholds of *R. songarica* seeds varied across the nine source locations, potentially reflecting the habitat conditions of the parent plant. Plants that grow in areas with abundant water may produce seeds that are genetically adapted to these conditions, leading to reduced drought resistance in the progeny and adaptations more suited to humid environments (Amusa et al. 2020; Thorogood et al. 2018). In contrast, the source locations S2 and S3, located in more arid regions, show that *R. songarica* is better adapted to drought‐prone habitats, with its reproductive organs exhibiting enhanced drought resistance to ensure population persistence and expansion even in harsh environments.

Studies on the growth of *R. songarica* seedlings under drought conditions revealed a significant decrease in RL and a rapid decline at specific points, but no such decline was observed in PL. This suggests that the root system, being the first organ to experience drought stress, is particularly sensitive to changes in drought intensity and exhibits distinctive responses (Tianzhen et al. 2024). This finding is further supported by the observed decline in the root‐crown ratio. In our study on drought‐resistant germination of *R. songarica* seeds, we found that low concentrations of PEG solutions promoted seed germination, although seedling growth was inhibited. The reduced abscisic acid levels in the seeds, coupled with increased gibberellin accumulation induced by the low PEG concentration, primarily contributed to the enhanced seed germination (Gurusinghe and Bradford 2001). However, the reduction in water potential led to greater water absorption by the seedlings, which inhibited their growth.

In summary, the effects of drought on the seed germination and seedling growth of *R. songarica* are multifaceted, encompassing both nutrient dynamics and enzyme activity. A combined analysis of phenotypic traits and intrinsic physiological responses could provide valuable insights into the mechanisms underlying drought‐resistant germination in *R. songarica*. While these aspects are beyond the scope of this study, they represent promising directions for future research.

## 5 Conclusion

This study investigated the seed traits, germination characteristics, and seedling growth of *R. songarica* from nine seed source locations at different harvesting periods. It was concluded that in the Hehuang Valley, the optimal harvesting period for *R. songarica* seeds ranges from 60 to 100 days after flowering. Furthermore, it is recommended to prioritize germplasm resources from arid seed source locations for seed harvesting. Subsequently, the seed traits, germination characteristics, seedling growth parameters, and drought tolerance of *R. songarica* were evaluated for the nine optimal harvesting periods, leading to the following conclusions: (1) Germination characteristics and seedling growth parameters of *R. songarica* showed significant variability across the seed source gradient, with seeds from arid source locations exhibiting higher germination potential and greater seedling development capacity. (2) Seed 1000‐seed weight and SV primarily influenced seed germination, while relative conductivity and absolute conductivity significantly determined seedling growth potential. (3) Structural equation modeling revealed that precipitation at the seed source location significantly affected seed 1000‐seed weight, while absolute conductivity and seed width had notable impacts on germination drought tolerance index, seed germination characteristics, and seedling growth state. This study not only elucidates the effects of seed source location on seed germination and seedling growth but also provides practical guidance for the selection, expansion, and application of *R. songarica* germplasm resources in desertification restoration efforts. It establishes a theoretical foundation for future research directions. In the future, field verification of drought tolerance of different *R. songarica* germplasms can be carried out based on the results of this study, which will be conducive to the application of *R. songarica* in practice.

## Author Contributions

**XinYou Wang:** conceptualization (lead), formal analysis (lead), investigation (lead), methodology (lead), software (lead), visualization (lead), writing – original draft (lead), writing – review and editing (lead). **Lijun Zhang:** conceptualization (supporting), investigation (supporting), methodology (supporting), software (supporting), supervision (supporting), validation (supporting). **Zhengshegn Li:** conceptualization (supporting), investigation (supporting), methodology (supporting), supervision (supporting), visualization (supporting). **Yanlong Wang:** investigation (equal), methodology (supporting), software (supporting), supervision (supporting), validation (supporting). **Ying Liu:** conceptualization (supporting), funding acquisition (equal), validation (supporting), writing – review and editing (equal). **YuShou Ma:** conceptualization (supporting), funding acquisition (lead), investigation (equal), supervision (lead), validation (supporting), writing – review and editing (equal).

## Conflicts of Interest

The authors declare no conflicts of interest.

## Supporting information


**Table S1:** Seed sampling timetable for *Reaumuria songarica*.
**Table S2:** Seed germination rate of *R. songarica* by batch.

## Data Availability

The data that support the findings of this study are openly available in Mendeley at https://data.mendeley.com/datasets/fyftn9wvtv/1.
