# Supplementary material for: Seed‐Source Precipitation Drives Drought Acclimatization of Reaumuria songarica: Implications for Seed Germination and Seedling Growth in the HeHuang Valley
Source: Ecol Evol. 2025 Aug 24;15(8):e72010. doi: 10.1002/ece3.72010 (PMC12375825; doi:10.1002/ece3.72010)
Supplement: Supplementary file 1 — Table S1: Seed sampling timetable for Reaumuria songarica. Table S2: Seed germination rate of R. songarica by batch. [file ECE3-15-e72010-s001.docx]

Table S1 Seed sampling timetable for *Reaumuria songarica*

| Sampling point | Sampling time (month-day) | | | | | | | | |
| --- | --- | --- | --- | --- | --- | --- | --- | --- | --- |
|  | T1 | T2 | T3 | T4 | T5 | T6 | T7 | T8 | T9 |
| S1 | 7-16 | 7-29 | 8-9 | 8-20 | 8-30 | 9-10 | 9-20 | 10-2 | 10-12 |
| S2 | 7-16 | 7-29 | 8-9 | 8-20 | 8-30 | 9-10 | 9-20 | 10-2 | 10-12 |
| S3 | 7-16 | 7-29 | 8-9 | 8-20 | 8-30 | 9-10 | 9-20 | 10-2 | 10-12 |
| S4 | 8-9 | 8-20 | 8-30 | 9-10 | 9-20 | 10-2 | 10-12 | 10-20 | — |
| S5 | 8-20 | 8-30 | 9-10 | 9-20 | 10-2 | 10-12 | 10-20 | — | — |
| S6 | 8-9 | 8-20 | 8-30 | 9-10 | 9-20 | 10-2 | 10-12 | 10-20 | — |
| S7 | 8-9 | 8-20 | 8-30 | 9-10 | 9-20 | 10-2 | 10-12 | 10-20 | — |
| S8 | 8-9 | 8-20 | 8-30 | 9-10 | 9-20 | 10-2 | 10-12 | 10-20 | — |
| S9 | 8-20 | 8-30 | 9-10 | 9-20 | 10-2 | 10-12 | 10-20 | — | — |

Note: All samples were collected in the year 2023, “—” means data not acquired, the same as below.

Table S2 Seed germination rate of *R. songarica* by batch

| Sampling point | Sampling time | | | | | | | | | |
| --- | --- | --- | --- | --- | --- | --- | --- | --- | --- | --- |
|  | T1 | T2 | T3 | T4 | T5 | T6 | T7 | T8 | T9 | T10 |
| S1 | 0.0000 ±  0.0000 | 0.4581 ±  0.0612 | 0.6772 ±  0.0499 | 0.8261 ±  0.0348 | 0.9731 ±  0.0124 | 0.9722 ±  0.0124 | 0.9213 ±  0.0069 | 0.9141 ±  0.0069 | 0.9353 ±  0.0306 | 1.0523 ±  0.0336 |
| S2 | 0.0000 ±  0.0000 | 0.2742 ±  0.0131 | 0.7321 ±  0.0177 | 1.1621 ±  0.0158 | 1.1611 ±  0.0158 | 0.92732 ±  0.0122 | 1.1221 ±  0.0493 | 1.1883 ±  0.0167 | 1.1081 ±  0.0755 | 1.0913 ±  0.0218 |
| S3 | 0.0000 ±  0.0000 | 0.3821 ±  0.0281 | 0.7123 ±  0.0178 | 0.9991 ±  0.0298 | 1.0753 ±  0.0079 | 0.9632 ±  0.0143 | 1.0441 ±  0.0204 | 1.0673 ±  0.0079 | 1.0313 ±  0.054 | 1.1015 ±  0.0358 |
| S4 | 0.5427 ±  0.0328 | 0.7053 ±  0.0311 | 0.6285 ±  0.0427 | 0.9253 ±  0.0069 | 0.9776 ±  0.0258 | 1.0515 ±  0.0077 | 1.0368 ±  0.0132 | 1.1088 ±  0.0145 | — | — |
| S5 | 0.4469 ±  0.0225 | 0.8194 ±  0.0067 | 0.6855 ±  0.0118 | 1.0843 ±  0.0363 | 1.0468±  0.0444 | 1.0216 ±  0.0075 | 0.9982 ±  0.0073 | — | — | — |
| S6 | 0.5359 ±  0.0132 | 0.7853 ±  0.0235 | 0.6712 ±  0.0298 | 0.7656 ±  0.0419 | 1.0135 ±  0.0129 | 1.0671 ±  0.0216 | 1.0226 ±  0.0329 | 1.0223 ±  0.0329 | — | — |
| S7 | 0.3215 ±  0.0193 | 0.6646 ±  0.0119 | 0.8599 ±  0.0244 | 1.0217 ±  0.0199 | 0.9784 ±  0.0316 | 0.9848 ±  0.0072 | 0.8054 ±  0.0231 | 0.9341 ±  0.0185 | — | — |
| S8 | 0.4416 ±  0.0607 | 0.7797 ±  0.0067 | 0.8395 ±  0.0356 | 0.8323 ±  0.0134 | 1.17234 ±  0.0414 | 1.0516 ±  0.0156 | 0.9143 ±  0.0183 | 0.9848 ±  0.0191 | — | — |
| S9 | 0.6717 ±  0.0181 | 0.5794 ±  0.0252 | 0.9493 ±  0.0325 | 0.9073 ±  0.0318 | 0.9026 ±  0.0571 | 1.0135 ±  0.0129 | 0.9481 ±  0.0122 | — | — | — |
